# Non-Dexamethasone Corticosteroid Therapy’s Effect on COVID-19 Prognosis in Cancer Patients: A Retrospective Study

**DOI:** 10.3390/vaccines11020290

**Published:** 2023-01-28

**Authors:** Lina Souan, Zienab Al-Khairy, Abdelkader Battah, Maher A. Sughayer

**Affiliations:** 1 Department of Pathology & Laboratory Medicine, King Hussein Cancer Center, Amman 11941, Jordan; 2 Department of Pathology, Microbiology and Forensic Medicine, School of Medicine, The University of Jordan, Amman 11942, Jordan

**Keywords:** COVID-19, cancer patients, hydrocortisone, prednisone, corticosteroid, therapy, SARS-CoV-2, ventilation assistance, admission to hospital, mortality, systemic corticosteroid

## Abstract

Background: Anti-inflammatory corticosteroids are used in cancer treatment and COVID-19 infections. Data on the impact of non-dexamethasone corticosteroids on COVID-19 infection severity in cancer patients are minimal. This study investigates if corticosteroid treatment affects the disease severity in adult cancer patients. Methods: A total of 116 COVID-19-infected cancer patients on hydrocortisone (H) or prednisone (P) were compared to 343 untreated patients. The study included patients who received corticosteroids before (B), after (A), or both before and after (B and A) COVID-19 infections. Ventilation support, hospitalization and mortality were investigated. Results: Our data showed that a significantly greater number of patients taking H or P required ventilation support and hospitalization and that mortality rates were higher than the control group. Patients who received H or P after COVID-19 infection had a significantly worse prognosis than the other sub-groups and the control group. Conclusion: Corticosteroids impacted cancer patients’ COVID-19 prognosis. Despite the limited sample size, H- and P-treated patients’ corticosteroids performed worse than the control, especially if treatments were received after COVID-19 infection. Hence, when a cancer patient already on H or P treatment is diagnosed with COVID-19, we recommend switching to a steroid treatment as suggested by international guidelines.

## 1. Introduction

In cancer treatment, corticosteroids may be administered as part of supportive care [1] or as part of treatment regimens. Corticosteroids are used to treat a variety of tumors and complications related to cancer treatment and promote apoptosis in hematological neoplasms [2,3,4]. They are also used to regulate anaphylaxis and cytokine release, hypersensitivity, and suppress anaphylaxis [5,6,7]. One study showed that using hydrocortisone, a type of corticosteroid, improved the quality-of-life parameters for pain control, specifically in hormone-refractory prostate cancer patients [8]. Due to the anti-inflammatory properties of corticosteroids, it has been proposed that they could be used to treat or decrease COVID-19 infection complications [9,10,11]. Numerous studies revealed that the use of corticosteroids on non-cancer patients was associated with a 28-day reduction in mortality [9] and an increase in the number of days spent without the assistance of mechanical ventilation, particularly in patients who were critically ill [9,11,12,13,14,15,16,17,18,19].

Recent research has demonstrated that patients with progressing cancer may be more susceptible to COVID-19 infection because of their immunocompromised status. This is because patients with active cancer already have weakened immune systems due to the treatment they receive for their cancer, the underlying cause of their illness, and the severity of their disease. This makes cancer patients more susceptible to contracting and dying from COVID-19 infection [20,21]. It was shown that patients suffering from thoracic and hematologic malignancies had worse COVID-19 infection outcomes; however, information about the consequences of COVID-19 infection in cancer patients treated with immunomodulatory drugs is still contradictory [22,23,24,25,26].

Dexamethasone therapy for COVID-19 patients was recently evaluated in a controlled, open-label, randomized trial. The results showed that SARS-CoV-2 patients treated with dexamethasone had a reduced 28-day mortality than those receiving invasive mechanical ventilation or oxygen alone [12]. Furthermore, our previous published data showed that cancer patients who were already taking dexamethasone and continued the treatment after being infected with SARS-CoV-2 had higher survival rates and a better prognosis for COVID-19 than patients who stopped the medication after being diagnosed with COVID-19 [27].

As a follow-up to our previous work, we decided to investigate the effects of other corticosteroid management plans on cancer patients infected with the SARS-CoV-2 virus [27]. Therefore, this study aims to investigate if concurrent hydrocortisone and prednisone administration within seven days, particularly before, after, or both before and after a COVID-19 infection, influences the severity indicators of COVID-19 disease in cancer patients.

## 2. Materials and Methods

### 2.1. Study Design

**Figure 1:**
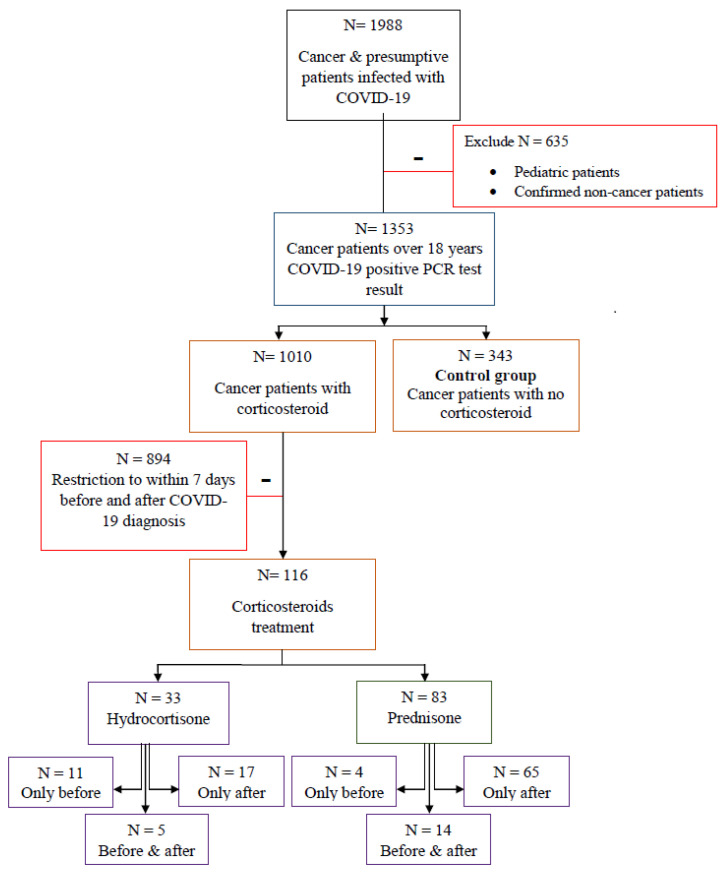
Methodology outline, including selection and rejection criteria.

For this study, we analyzed data from the medical records of COVID-19-infected cancer patients at King Hussein Cancer Center (KHCC) who were between 18 and 91 years old. All KHCC patients were Middle Eastern (including Jordanians) and followed KHCC’s treatment protocols [27]. The KHCC Cancer Registry records were searched for PCR-positive COVID-19 patients (N = 1988). After excluding children, non-cancer patients, and duplicates, 1353 PCR-positive COVID-19 adult cancer patients’ tests remained. Three hundred and forty-three PCR-positive COVID-19 adult cancer patient tests and no history of corticosteroids were used as the control group. The remaining 1010 cancer patients received corticosteroids for various indications. We limited corticosteroid therapy to hydrocortisone or prednisone and the treatment window to seven days before or after a positive COVID-19 result, according to Jee and colleagues’ recent study [17]. Therefore, the number of hydrocortisone and prednisone-treated cancer patients who also had COVID-19 infection within seven days dropped to 116 (Figure 1).

When classifying the various forms of cancer, the primary categories utilized were those pertaining to solid tumors and hematological malignancies. In the category of solid tumors, practically every organ was covered by at least one example of cancer. It was primarily distributed based on the most common forms (lung, breast, and colorectal cancer), while at the same time various forms of leukemia, lymphoma, and multiple myeloma were included as hematological cancers. Whether corticosteroids are used as a treatment or adjuvant therapy, the patient’s overall health, the type of cancer, and the chemotherapy regimen all have a role in determining the appropriate dosage. Hydrocortisone doses ranged between 20 and 240 mg, whereas prednisone ranged between 10 and 100 mg.

### 2.2. Patient Subgroups Based on the Timing of the COVID-19 Infection and Their Hydrocortisone Treatment Regimen

Hydrocortisone treatment groups were divided into three subgroups based on when hydrocortisone was given concerning the COVID-19 diagnostic. The first grouping was cancer patients who were using hydrocortisone within seven days of a positive COVID-19 PCR test but “stopped” taking it after the diagnosis. HcB stands for “hydrocortisone before”. Second, cancer patients started hydrocortisone within seven days after a positive COVID-19 PCR test. HcA stands for “hydrocortisone treatment after”. The third category included patients taking hydrocortisone medication within seven days before a positive COVID-19 PCR test result and taking it for at least seven days afterward. HcBA stands for “Hydrocortisone before and after”.

### 2.3. Outcome and Survival of the Three Hydrocortisone Subgroups

We compared the three hydrocortisone-treated subgroups to the control group using nonparametric tests, and the indicator variables were either the need for mechanical ventilation, hospitalization, or admission to the ICU, or mortality within 28 days of COVID-19 infection. In the inter-group analysis, these disease severity indicator variables were also used.

### 2.4. Subgroups of Patients by Prednisone Therapy and COVID-19 Onset

The prednisone treatment groups were divided into three subgroups based on when prednisone was given in relation to the COVID-19 infection. The first grouping was cancer patients who were using prednisone within seven days of a positive COVID-19 PCR test but “stopped” taking it after the diagnosis. PDN-B stands for “prednisone before”. Second, cancer patients started prednisone within seven days after a positive COVID-19 PCR test. PDN-A stands for “prednisone treatment after”. The third category included patients taking prednisone medication within seven days before a positive COVID-19 PCR test result and continuing to take it for at least seven days afterward. PDN-BA stands for “Prednisone before and after”.

### 2.5. Outcome and Survival of the Three Prednisone Subgroups

Using nonparametric testing, we compared the three prednisone-treated subgroups to the control group; the indicator variables were the need for mechanical breathing, hospitalization or ICU admission, or mortality within 28 days of COVID-19 infection. In the inter-group analysis, these indicator variables for disease severity were also applied.

### 2.6. Statistical Analysis

To analyze the data of SARS-CoV-2 infected patients who were treated between September 2020 and January 2022, we followed the same methodology as in our previous publication [27]. Software “R” version R 4.1.2 (2021) was used for all data manipulation and statistical analysis [28].

## 3. Results

### 3.1. Treatment with Non-Dexamethasone Corticosteroids and the Outcome of COVID-19 Disease

This study included a total of 459 adult cancer patients who were diagnosed with SARS-CoV-2 in the period from September 2020 to January 2022. The mean age was 55.8 years (SD 15.3, median 57). The females were the dominant (242/451, 53.66%). Most patients had solid tumors (408/459, 90.69%). Demographic characteristics are listed in Table 1.

Regarding clinical characteristics, in Table 2, of these participants, we observed a significantly higher percentage of patients who needed ventilation assistance in both the hydrocortisone group (14/33, 42%) and the prednisone group (48/83, 58%) compared to the control group (7/343, 2%, *p*-value < 0.05). Hospital admission is significantly higher in the hydrocortisone group (30/33, 91%) and prednisone group (71/83, 86%) than in the control group (59/343, 17%, *p*-value < 0.05). Similarly, admission to ICU in both groups (hydrocortisone: 16/33, 48%, and prednisone: 43/83, 52%) was significantly higher than the control group (2/341, 1%, *p*-value < 0.05). Finally, the mortality rate is higher in both groups (hydrocortisone: 19/33, 58%, and prednisone: 54/83, 65%) than in the control group (36/343, 10%, *p*-value < 0.05). Indicator variables are listed in Table 2.

### 3.2. Comparison between the Three Hydrocortisone-Treated Subgroups and the Control Group

Compared to the control group, all three subgroups, namely HcB, HcA, and HcBA, had significantly higher rates of requiring ventilator support, hospital, and ICU admission and had a higher mortality rate within 28 days (*p*-value < 0.05) (Table 3).

### 3.3. Hydrocortisone Inter-Subgroup Comparison

The COVID-19 severity indicator variables were used to compare the three subgroups. Table 4 shows no statistically significant difference between the three hydrocortisone subgroups except in admission to the ICU where the HcA subgroup showed a significantly worse prognosis compared to the HcBA subgroup (*p* < 0.05).

### 3.4. Comparison between the Three Prednisone Subgroups and the Control Group

A total of 83 patients received prednisone treatment. Compared to the control group, patients in the PDN-B subgroup showed no significant differences in any of the indicators, which might be due to the small sample size of this subgroup (only four patients). As for the second subgroup, patients who received prednisone therapy after COVID-19 infection, our data showed that this subgroup (PDN-A) had a significant increase in the four indicators (ventilation assistance, admission to hospital, admission to ICU, and mortality rate) when compared to the control group (*p*-value < 0.05). Similarly, the subgroup of patients who had prednisone treatment before and after COVID-19 infection had a significant increase in ventilation assistance, admission to hospital and ICU, and mortality rate in comparison to the control group (*p*-value < 0.05) (Table 5).

### 3.5. Prednisone Inter-Subgroup Comparison

When we compared the three prednisone subgroups, we found that the group of patients who were given prednisone before COVID-19 diagnosis (PDN-B) had significantly less ventilation assistance need compared to PDN-A and PDN-BA subgroups. Moreover, the PDN-B subgroup had fewer mortality rates compared to the PDN-BA subgroup (Table 6). This observation might be because of the small group size in the PDN-B subgroup, which consisted of only four patients. Interestingly, the PDN-BA subgroup patients had significantly higher admission rates to the ICU compared to the PDN-A subgroup (Table 6).

## 4. Discussion

In this study, we showed that non-dexamethasone corticosteroids might significantly affect the severity and outcome of COVID-19 infection. Our findings revealed that hydrocortisone and prednisone had a significantly worse outcome in the indicator variables studied (need for ventilation assistance, admission to hospital, admission to ICU, and mortality rates within 28 days of COVID-19 infection) compared to the control group. These indicators were significantly better in the control group compared to the non-dexamethasone corticosteroid-treated cancer patients.

Considering that patients in the control group did not receive any corticosteroids prior to or after their COVID-19 diagnosis, this might have been an indirect indicator that this group of patients was in a healthier state than those in the corticosteroid-treated groups. However, the demographics were similar between the control and the study groups except for more representation of hematological malignancies in the study group. We also divided the corticosteroid-treated patients into three subgroups for each treatment regimen. We then compared these subgroups to the control group and one another.

Through a detailed intra-subgroup analysis, we found that the three subgroups of hydrocortisone had a significantly increased rate of ventilation assistance, hospital and ICU admission, and increased mortality rate compared to the control group *p* < 0.05. Moreover, the HcA subgroup of patients showed a significantly higher ICU admission than the HcBA subgroup. This may mean that a cancer patient with a COVID-19 infection should keep receiving hydrocortisone treatment instead of starting hydrocortisone treatment after the infection. Alternatively, switching the corticosteroid type to dexamethasone would be preferable, as the REMAP-CAP randomized study group [29] and the RECOVERY study group recommended [30]. This conclusion is similar to our previous study on COVID-19-infected cancer patients treated with dexamethasone, which found that it is preferable to continue the corticosteroid rather than stop it [27].

As for prednisone, single variable analysis of the data showed that PDN-A patient subgroup and PDN-BA subgroup had significantly increased rates of ventilation assistance, admission to hospital and ICU, and mortality within 28 days of the COVID-19 diagnosis (*p*-value < 0.05) compared to the control group. This finding is consistent with Marte et al.’s study [31], which found that early prednisone administration might impair essential antiviral subsets and soluble factors leading to increased mortality.

Interestingly our data demonstrated that the PDN-BA subgroup had a significantly worse prognosis than PDN-A in requiring ventilation assistance and showed significantly higher mortality rates. These data may suggest that if a cancer patient is diagnosed with SARS-CoV-2 infection, it is preferable to discontinue prednisone treatment and switch to another corticosteroid (such as dexamethasone) rather than continuing prednisone treatment after the disease.

Although our study is the first retrospective-interim analysis of cancer patients who were already taking non-dexamethasone corticosteroids within seven days prior to COVID-19 disease or started the steroids within seven days from SARS-CoV-2 infection as a supportive care co-medication to reduce the unfavorable effects of anticancer therapies, our analysis has limitations which stem from the very structure of the method itself. The limited number of participants in each category hampered our ability to conduct robust statistical analyses and draw firm findings in many instances. Another problem was that there was no comparison of corticosteroid doses between the before and after groups. Because other clinical variables of the enrolled patients, such as cancer stage, ongoing cancer treatments, and comorbidities, were unavailable, a comparison between cancer patients and a control group with and without steroid therapy was not possible. Hence, a comprehensive prospective randomized controlled trial comparing early and late corticosteroid administration is required to precisely determine optimal corticosteroid administration timing in COVID-19-infected cancer patients. This is because the probability that cancer patients in the control group may be healthier might have influenced the results in this study.

## 5. Conclusions

We found that both corticosteroids, hydrocortisone and prednisone, had significantly higher rates of ventilation assistance, admission to hospital and/or ICU, and mortality rates within 28 days of COVID-19 compared to the control group (*p*-value < 0.05) regardless of the treatment regimen. Moreover, our data showed that the probability of death or the requirement for mechanical ventilator assistance within 28 days of infection was higher in individuals who received hydrocortisone or prednisone only after COVID-19 infection. This result was in contrast to the effect of dexamethasone therapy, which suggested that taking the medication after the SARS-CoV-2 infection or continuing it after COVID-19 disease may have more favorable effects on COVID-19 prognosis in cancer patients [27]. Finally, we conclude that treatment with hydrocortisone or prednisone worsens the COVID-19 prognosis in cancer patients compared to the control group. In addition, we recommend that cancer patients diagnosed with COVID-19 infection might switch to steroid treatment as suggested by international guidelines.

## Figures and Tables

**Table 1:** Sample characteristics according to treatment arm.

	Control	Hydrocortisone		Prednisone	
	N (%)	N (%)	*p*-Value	N (%)	*p*-Value
**Total (N = 459)**	343 (74.7%)	33 (7.2%)		83 (18.1%)	
**Age Range (years)**	20–89	19–85		23–87	
**Median age (years)**	58.0	61.0	0.350	61.0	0.095
**Sex**					
Male	151 (44%)	19 (58%)	0.190	48 (58%)	0.324
Female	192 (56%)	14 (42%)		35 (42%)	
**Cancer type**					
Solid (N = 408)	318 (93%)	24 (75%)	<0.05	66 (80%)	<0.05
Hematological (N = 49)	25 (7%)	8 (25%)		16 (20%)	

N: sample number. Patients with unknown cancer primary sites were excluded from these percentages.

**Table 2:** Indicator variables of the study population divided by the study groups.

	Control	Hydrocortisone		Prednisone	
	N (%)	N (%)	*p*-Value	N (%)	*p*-Value
**Total (N = 459)**	343 (74.7%)	33 (7.2%)		83 (18.1%)	
**Ventilation assistance**					
Yes	7 (2%)	14 (42%)	<0.05	48 (58%)	<0.05
No	336 (98%)	19 (58%)		35 (42%)	
**Admitted to hospital**					
Yes	59 (17%)	30 (91%)	<0.05	71 (86%)	<0.05
No	284 (83%)	3 (9%)		12 (14%)	
**Admitted to ICU**					
Yes	2 (1%)	16 (48%)	<0.05	43 (52%)	<0.05
No	341 (99%)	17 (52%)		40 (48%)	
**Mortality within 28 days of COVID-19**					
Yes	36 (10%)	19 (58%)	<0.05	54 (65%)	<0.05
No	307 (90%)	14 (42%)		29 (35%)	

N: sample number. Patients with unknown cancer primary site were excluded from these percentages.

**Table 3:** Summary of variable values for control and hydrocortisone groups.

	Control	Treated Only before COVID-19 Diagnosis		Treated Only after COVID-19 Diagnosis		Treated before and after COVID-19 Diagnosis	
Indicators Variables	N (%)	N (%)	*p*-Value **	N (%)	*p*-Value **	N (%)	*p*-Value **
**Total (N = 376)**	343 (91.2%)	11 (2.9%)		17 (4.5%)		5 (1.3%)	
**Ventilation assistance**							
Yes	7 (2%)	4 (36%)	<0.05	8 (47%)	<0.05	2 (40%)	<0.05
No	336 (98%)	7 (64%)		9 (53%)		3 (60%)	
**Admitted to hospital**							
Yes	59 (17%)	8 (73%)	<0.05	17(100%)	<0.05	5 (100%)	<0.05
No	284 (83%)	3 (27%)		0 (0%)		0 (0%)	
**Admitted to ICU**							
Yes	2 (1%)	5 (45%)	<0.05	10 (59%)	<0.05	1 (20%)	<0.05
No	341 (99%)	6 (55%)		7 (41%)		4 (80%)	
**Mortality within 28 days of COVID-19**							
Yes	36 (10%)	6 (55%)	<0.05	10 (59%)	<0.05	3 (60%)	<0.05
No	307 (90%)	5 (45%)		7 (41%)		2 (40%)	

All *p*-values represent the results of testing differences between each medicated group and the control group. *p*-value **: *p*-value from Fisher’s exact test or Pearson’s chi-square test where appropriate. All *p*-values in the table are corrected for multiple testing using the Benjamini–Hochberg method. SD: standard deviation, IQR: interquartile range.

**Table 4:** Effect of hydrocortisone treatment before and after COVID-19 infection on disease indicators.

Groups	Hydrocortisone Treatment before vs, after COVID-19 Infection		Hydrocortisone Treatment before vs. before and after COVID-19 Infection		Hydrocortisone Treatment after vs. before and after COVID-19 Infection	
Indicators Variables	HcB vs. HcA	*p*-Value	HcB vs. HcBA	*p*-Value	HcA vs. HcBA	*p*-Value
	N (%)		N (%)		N (%)	
Ventilation assistance	4 (36%) vs. 8 (47%)	0.867	4 (36%) vs. 2 (40%)	1	8 (47%) vs. 2 (40%)	1
Admitted to the hospital	8 (73%) vs. 17 (100%)	0.0983	8 (73%) vs. 5 (100%)	0.546	17 (100%) vs. 5 (100%)	1
Admitted to ICU	5 (45%) vs. 10 (59%)	0.761	5 (45%) vs. 1 (20%)	0.676	10 (59%) vs. 1 (20%)	0.024
Mortality within 28 days of COVID-19	6 (55%) vs. 10 (59%)	1	6 (55%) vs. 3 (60%)	1	10 (59%) vs. 3 (60%)	1

N: number of patients. *p*-value: represent the results of testing differences between each subgroup and the other. Fisher’s exact test or chi-square test was used as appropriate.

**Table 5:** Summary of variable values for control and prednisone groups.

Prednisone Groups	Control	Only before COVID-19 within 7 Days		Only after COVID-19 within 7 Days		Before and after COVID-19 within 7 Days	
Indicators Variables	N (%)	N (%)	*p*-Value **	N (%)	*p*-Value **	N (%)	*p*-Value **
**Total (N = 426)**	343 (80.5%)	4 (0.9%)		65 (15.3%)		14 (3.5%)	
**Ventilation assistance**							
Yes	7 (2%)	0 (0%)	1	37 (57%)	<0.05	11 (79%)	<0.05
No	336 (98%)	4 (100%)		28 (43%)		3 (21%)	
**Admitted to hospital**							
Yes	59 (17%)	3 (75%)	0.0554	55 (85%)	<0.05	13 (93%)	<0.05
No	284 (83%)	1 (25%)		10 (15%)		1 (7%)	
**Admitted to ICU**							
Yes	2 (1%)	1 (25%)	0.0955	31 (48%)	<0.05	11 (79%)	<0.05
No	341 (99%)	3 (75%)		34 (52%)		3 (21%)	
**Mortality within 28 days of COVID-19**							
Yes	36 (10%)	1 (25%)	0.580	41 (63%)	<0.05	12 (86%)	<0.05
No	307 (90%)	3 (75%)		24 (37%)		2 (14%)	

All *p*-values represent the results of testing differences between each medicated group and the control group. *p*-value **: *p*-value from Fisher’s exact test or Pearson’s chi-square test where appropriate. All *p*-values in the table are corrected for multiple testing using the Benjamini–Hochberg method. SD: standard deviation, IQR: interquartile range.

**Table 6:** Effect of prednisone treatment before, after, and before and after COVID-19 infection on disease indicators.

Groups	Prednisone Treatment before vs after COVID-19 Infection			Prednisone Treatment before vs before and after COVID-19 Infection			Prednisone Treatment after vs before and after COVID-19 Infection		
Indicators Variables	PDN B vs. PDN A		*p*-Value	PDN B vs. PDN BA		*p*-Value	PDN A vs. PDN BA		*p*-Value
	N (%)			N (%)			N (%)		
**Ventilation assistance**									
Yes	0 (0%)	37 (57%)	0.042 *	0 (0%)	11 (79%)	0.011 *	37 (57%)	11 (79%)	0.132 **
No	4 (100%)	28 (43%)		4 (100%)	3 (21%)		28 (43%)	3 (21%)	
**Admitted to the hospital**									
Yes	3 (75%)	55 (85%)	0.509 *	3 (75%)	13 (93%)	0.405 *	55 (85%)	13 (93%)	0.419 **
No	1 (25%)	10 (15%)		1 (25%)	1 (7%)		10 (15%)	1 (7%)	
**Admitted to ICU**									
Yes	1 (25%)	31 (48%)	0.618 *	1 (25%)	11 (79%)	0.083 *	31 (48%)	11 (79%)	0.036 **
No	3 (75%)	34 (52%)		3 (75%)	3 (21%)		34 (52%)	3(21%)	
**Mortality within 28 days of COVID-19**									
Yes	1 (25%)	41 (63%)	0.292 *	1 (25%)	12 (86%)	0.044 *	41 (63%)	12 (86%)	0.102 **
No	3 (75%)	24 (37%)		3 (75%)	2 (14%)		24 (37%)	2 (14%)	

* denotes the use of Fisher’s exact test where we used it if >40% have expected count < 5. ** denotes the use of chi-square test.

## Data Availability

The data presented in this study are available by request from the corresponding author. The data are not publicly available for ethical reasons.

## Abbreviations

SARS-CoV-2 (Severe Acute Respiratory Syndrome Coronavirus 2), ICU (intensive care unit), PCR (polymerase chain reaction), FEU (fibrinogen equivalent units).

## References

[1] Chang C.W.D., McCoul E.D., Briggs S.E., Guardiani E.A., Durand M.L., Hadlock T.A., Hillel A.T., Kattar N., Openshaw P.J., Osazuwa-Peters N. (2022). Corticosteroid Use in Otolaryngology: Current Considerations During the COVID-19 Era. Otolaryngol. Neck Surg..

[2] Herr I., Büchler M.W., Mattern J. (2009). Glucocorticoid-Mediated Apoptosis Resistance of Solid Tumors. Death Recept. Cogn. Ligands Cancer.

[3] Pufall M.A. (2015). Glucocorticoids and Cancer. Glucocorticoid Signal..

[4] Samueli B., Nalbandyan K., Benharroch D., Levi I. (2021). Splenic Micronodular T-Cell/Histiocyte-Rich Large B-Cell Lymphoma: The Corticosteroid Pretreatment Hypothesis. Acta Haematol..

[5] Faggiano A., Mazzilli R., Natalicchio A., Adinolfi V., Argentiero A., Danesi R., D’Oronzo S., Fogli S., Gallo M., Giuffrida D. (2022). Corticosteroids in oncology: Use, overuse, indications, contraindications. An Italian Association of Medical Oncology (AIOM)/Italian Association of Medical Dia-betologists (AMD)/Italian Society of Endocrinology (SIE)/Italian Society of Pharmacology (SIF) multidisciplinary con-sensus position paper. Crit. Rev. Oncol. /Hematol..

[6] Aldea M., Orillard E., Mansi L., Marabelle A., Scotte F., Lambotte O., Michot J.M. (2020). How to manage patients with corticosteroids in oncology in the era of immunotherapy?. Eur. J. Cancer.

[7] Frieze D.A. (2010). Musculoskeletal Pain Associated With Corticosteroid Therapy in Cancer. Curr. Pain Headache Rep..

[8] Kantoff P.W., Halabi S., Conaway M., Picus J., Kirshner J., Hars V., Trump D., Winer E.P., Vogelzang N.J. (1999). Hydrocortisone With or Without Mitoxantrone in Men With Hormone-Refractory Prostate Cancer: Results of the Cancer and Leukemia Group B 9182 Study. J. Clin. Oncol..

[9] Liu J., Zhang S., Dong X., Li Z., Xu Q., Feng H., Cai J., Huang S., Guo J., Zhang L. (2020). Corticosteroid treatment in severe COVID-19 patients with acute respiratory distress syndrome. J. Clin. Investig..

[10] Patel V.K., Shirbhate E., Patel P., Veerasamy R., Sharma P.C., Rajak H. (2021). Corticosteroids for treatment of COVID-19: Effect, evidence, expectation and extent. Beni-Suef Univ. J. Basic Appl. Sci..

[11] Wagner C., Griesel M., Mikolajewska A., Mueller A., Nothacker M., Kley K., Metzendorf M.I., Fischer A.L., Kopp M., Stegemann M. (2021). Systemic corticosteroids for the treatment of COVID-19. Cochrane Database Syst. Rev..

[12] Group R.C., Horby P., Lim W.S., Emberson J.R., Mafham M., Bellm J.L., Staplin N., Brightling C., Ustianowski A., Elmahi E. (2021). Dexamethasone in Hospitalized Patients with COVID-19. N. Engl. J. Med..

[13] Matthay M.A., Wick K.D. (2020). Corticosteroids, COVID-19 pneumonia, and acute respiratory distress syndrome. J. Clin Investig..

[14] Sterne J.A., Murthy S., Diaz J.V., Slutsky A.S., Villar J., Angus D.C., Annane D., Azevedo L.C.P., Berwanger O., Cavalcanti A.B. (2020). Association Between Administration of Systemic Corticosteroids and Mortality among Critically Ill Patients with COVID-19: A Meta-analysis. JAMA.

[15] Tomazini B.M., Maia I.S., Cavalcanti A.B., Berwanger O., Rosa R.G., Veiga V.C., Avezum A., Lopes R.D., Bueno F.R., Silva M.V.A. (2020). Effect of Dexamethasone on Days Alive and Ventilator-Free in Patients With Moderate or Severe Acute Respiratory Distress Syndrome and COVID-19: The CoDEX Randomized Clinical Trial. JAMA.

[16] Guo Y.-R., Cao Q.-D., Hong Z.-S., Tan Y.-Y., Chen S.-D., Jin H.-J., Tan K.-S., Wang D.-Y., Yan Y. (2020). The origin, transmission and clinical therapies on coronavirus disease 2019 (COVID-19) outbreak—An update on the status. Mil. Med. Res..

[17] Jee J., Stonestrom A.J., Devlin S., Nguyentran T., Wills B., Narendra V., Foote M.B., Lumish M., Vardhana S.A., Pastores S.M. (2021). Oncologic immunomodulatory agents in patients with cancer and COVID-19. Sci. Rep..

[18] Karunasagar I., Karunasagar I. (2020). Ongoing COVID-19 global crisis and scientific challenges. J. Health Allied Sci. NU.

[19] Mungroo M.R., Khan N.A., Siddiqui R. (2020). The increasing importance of the novel Coronavirus. Hosp. Pr..

[20] Han H.J., Nwagwu C., Anyim O., Ekweremadu C., Kim S. (2021). COVID-19 and cancer: From basic mechanisms to vaccine devel-opment using nanotechnology. Int. Immunopharmacol..

[21] Rai P., Kumar B.K., Deekshit V.K., Karunasagar I., Karunasagar I. (2021). Detection technologies and recent developments in the diagnosis of COVID-19 infection. Appl. Microbiol. Biotechnol..

[22] Dai M.-Y., Liu D.-B., Liu M., Zhou F.-X., Li G.-L., Chen Z., Zhang Z.-A., You H., Wu M., Zheng Q.-C. (2020). Abstract CT406: Patients with cancer appear more vulnerable to SARS-COV-2: A multi-center study during the COVID-19 outbreak. Cancer Res..

[23] Kuderer N.M., Choueiri T.K., Shah D.P., Shyr Y., Rubinstein S.M., Rivera D.R., Shete S., Hsu C.-Y., Desai A., de Lima Lopes G. (2020). Clinical impact of COVID-19 on patients with cancer (CCC19): A cohort study. Lancet.

[24] Lee L.Y.W., Cazier J.-B., Angelis V., Arnold R., Bisht V., Campton N.A., Chackathayil J., Cheng V.W.T., Curley H.M., Fittall M.W.T. (2020). COVID-19 mortality in patients with cancer on chemotherapy or other anticancer treatments: A prospective cohort study. Lancet.

[25] Mehta V., Goel S., Kabarriti R., Cole D., Goldfinger M., Acuna-Villaorduna A., Pradhan K., Thota R., Reissman S., Sparano J.A. (2020). Case Fatality Rate of Cancer Patients with COVID-19 in a New York Hospital System. Cancer Discov..

[26] Yang K., Sheng Y., Huang C., Jin Y., Xiong N., Jiang K., Lu H., Liu J., Yang J., Dong Y. (2020). Clinical characteristics, outcomes, and risk factors for mortality in patients with cancer and COVID-19 in Hubei, China: A multicentre, retrospective, cohort study. Lancet Oncol..

[27] Souan L., Al-Khairy Z., Al-Binni M.A., Battah A., Sughayer M.A. (2022). The Effect of Dexamethasone Treatment on COVID-19 Prognosis in Cancer Patients. Vaccines.

[28] Team R.C. (2020). R: A Language and Environment for Statistical Computing.

[29] Angus D.C., Derde L., Al-Beidh F., Annane D., Arabi Y., Beane A., van Bentum-Puijk W., Berry L., Bhimani Z., Bonten M. (2020). Effect of Hydrocortisone on Mortality and Organ Support in Patients With Severe COVID-19: The REMAP-CAP COVID-19 Corticosteroid Domain Randomized Clinical Trial. JAMA.

[30] Horby P., Lim W., Emberson J., Mafham M., Bell J. (2021). Dexamethasone in Hospitalized Patients with Covid-19-Preliminary Report. N. Engl. J. Med..

[31] Marté J.L., Toney N.J., Cordes L., Schlom J., Donahue R.N., Gulley J.L. (2020). Early changes in immune cell subsets with corticosteroids in patients with solid tumors: Implications for COVID-19 management. J. Immunother. Cancer.

